# Isolation of a Rickettsial Pathogen from a Non-Hematophagous Arthropod

**DOI:** 10.1371/journal.pone.0016396

**Published:** 2011-01-25

**Authors:** Chutima Thepparit, Piyanate Sunyakumthorn, Mark L. Guillotte, Vsevolod L. Popov, Lane D. Foil, Kevin R. Macaluso

**Affiliations:** 1 Department of Pathobiological Sciences, School of Veterinary Medicine, Louisiana State University, Baton Rouge, Louisiana, United States of America; 2 Department of Pathology, WHO Collaborating Center for Tropical Diseases, University of Texas Medical Branch, Galveston, Texas, United States of America; 3 Department of Entomology, Louisiana State University, Baton Rouge, Louisiana, United States of America; National Institute of Allergy and Infectious Diseases, National Institutes of Health, United States

## Abstract

Rickettsial diversity is intriguing in that some species are transmissible to vertebrates, while others appear exclusive to invertebrate hosts. Of particular interest is *Rickettsia felis*, identifiable in both stored product insect pests and hematophagous disease vectors. To understand rickettsial survival tactics in, and probable movement between, both insect systems will explicate the determinants of rickettsial pathogenicity. Towards this objective, a population of *Liposcelis bostrychophila*, common booklice, was successfully used for rickettsial isolation in ISE6 (tick-derived cells). Rickettsiae were also observed in *L. bostrychophila* by electron microscopy and in paraffin sections of booklice by immunofluorescence assay using anti-*R. felis* polyclonal antibody. The isolate, designated *R. felis* strain LSU-Lb, resembles typical rickettsiae when examined by microscopy. Sequence analysis of portions of the *Rickettsia* specific 17-kDa antigen gene, citrate synthase (*gltA*) gene, rickettsial outer membrane protein A (*ompA*) gene, and the presence of the *R. felis* plasmid in the cell culture isolate confirmed the isolate as *R. felis*. Variable nucleotide sequences from the isolate were obtained for *R. felis*-specific pRF-associated putative *tldD*/*pmbA*. Expression of rickettsial outer membrane protein B (OmpB) was verified in *R. felis* (LSU-Lb) using a monoclonal antibody. Additionally, a quantitative real-time PCR assay was used to identify a significantly greater median rickettsial load in the booklice, compared to cat flea hosts. With the potential to manipulate arthropod host biology and infect vertebrate hosts, the dual nature of *R. felis* provides an excellent model for the study of rickettsial pathogenesis and transmission. In addition, this study is the first isolation of a rickettsial pathogen from a non-hematophagous arthropod.

## Introduction

The complexity of intracellular bacteria belonging to the genus *Rickettsia* is becoming more apparent as many species are being recognized as pathogens or endosymbionts of arthropods of medical or veterinary importance, seemly ubiquitous in many non-hematophagous insect pests, or both. Of particular interest in this mix of species is *Rickettsia felis*, which appears to be distinctly dualistic in nature. As a human pathogen, *R. felis* infection manifests itself in clinical symptoms typical for rickettsial infections (reviewed in [1]). Known to infect hematophagous arthropods, *R. felis* is primarily associated with cat fleas, *Ctenocephalides felis*. Experimental data suggest *R. felis* horizontal transmission by cat fleas to vertebrate hosts [2], although the model vertebrate host and route of horizontal transmission has yet to be clearly defined.

In addition to the relationships between pathogenic rickettsial species and hematophagous arthropods directly affecting human health, non-pathogenic rickettsial endosymbionts have been identified in annelids, amoebae, plants, and a variety of insects [3], [4]. Within the invertebrate host, many obligate endosymbionts such as *Rickettsia* in booklice, aphids, and bedbugs [5]–[7], *Rhizobium* in leeches [8], and *Buchnera* in aphids [9] reside in a specialized organ called a mycetome. The mycetome consists of clustering mycetocytes containing bacteria and may become an integral part of the host, sometimes essential for host fitness and fecundity [6], [7], [10]. For many of these *Rickettsia*/host relationships currently not associated with human disease the *Rickettsia* sp. has only been minimally examined through 16S rRNA gene molecular characterization [3], [4]. In *Liposcelis* spp. (booklice), *Rickettsia* manipulate reproductive biology inducing parthenogenesis [6] and are considered essential to optimal oviposition [11]. As an obligate endosymbiont of *Liposcelis bostrychophila*, rickettsiae target reproductive cells and have a dynamic cell association during insect development demonstrating a relatively synchronized relationship in this arthropod host [6].

Phylogenetic analysis places *R. felis* into the newly ascribed transitional group of *Rickettsia* [12]. This classification was recently confirmed by the placement of flea-associated *R. felis* in the transitional group, adjacent to the rickettsial species associated with *L. bostrychophila* [4]. Molecular characterization of rickettsiae infecting *L. bostrychophila* identified the agent as *R. felis* by matching a bacterial plasmid present in insects with that described in the cat flea isolate of *R. felis* [13]. The identification of *R. felis* in common non-hematophagous insects suggests a complex ecology, and isolation of this organism will facilitate the study of transmission routes.

Recently generated isolates of *R. felis* from fleas [14]–[16] further the appreciation of rickettsial biology including the identification of plasmid [17], strain variation of plasmid content [18], and characterization of a viable extracellular state [19]. The current study utilizes molecular and microscopic techniques to characterize, and the tick-derived ISE6 cell line to isolate, rickettsiae from *L. bostrychophila*. Microscopic analysis of the cultured organisms identified typical rickettsial morphology by histological staining and bright field microscopy and transmission electron microscopy (TEM). Genetic analysis of the *Rickettsia* specific 17-kDa antigen gene, citrate synthase (*gltA*) gene, rickettsial outer membrane protein A (*ompA*) gene, and rickettsial plasmid nucleotide sequences confirmed the isolate as *R. felis*. We subsequently used quantitative real-time PCR (qPCR) to estimate rickettsial load in *L. bostrychophila* and compare infection density between the cat flea and the booklouse. The close association between the cat fleas, booklice, and the vertebrate host; the availability of both the cat flea isolate (*R. felis* LSU) and the new isolate (*R. felis* LSU-Lb); and, colonized *Rickettsia*-infected arthropods, will provide a novel system to examine rickettsial pathogenesis and transmission.

## Results

### Detection of *Rickettsia* in booklice

**Figure 1:**
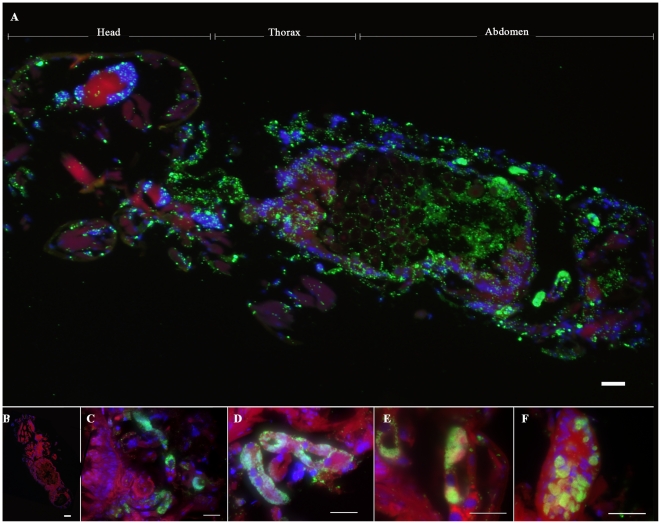
Detection of *Rickettsia* in booklice. (A) *Rickettsia* on formalin-fixed paraffin-embedded booklice sections were labeled with mouse polyclonal antibody against *R. felis* followed by FITC-conjugated goat anti-mouse IgG. Whole booklice tissues were counterstained with Evan's blue and nuclei were stained with DAPI as shown in red and blue, respectively. (B) Negative control staining using non-infected mouse serum. (C)–(F) Variation of mycetomes located in booklice abdomen. High magnification view of mycetomes shows densely packed rickettsiae. Bar = 100 µm.

**Figure 2:**
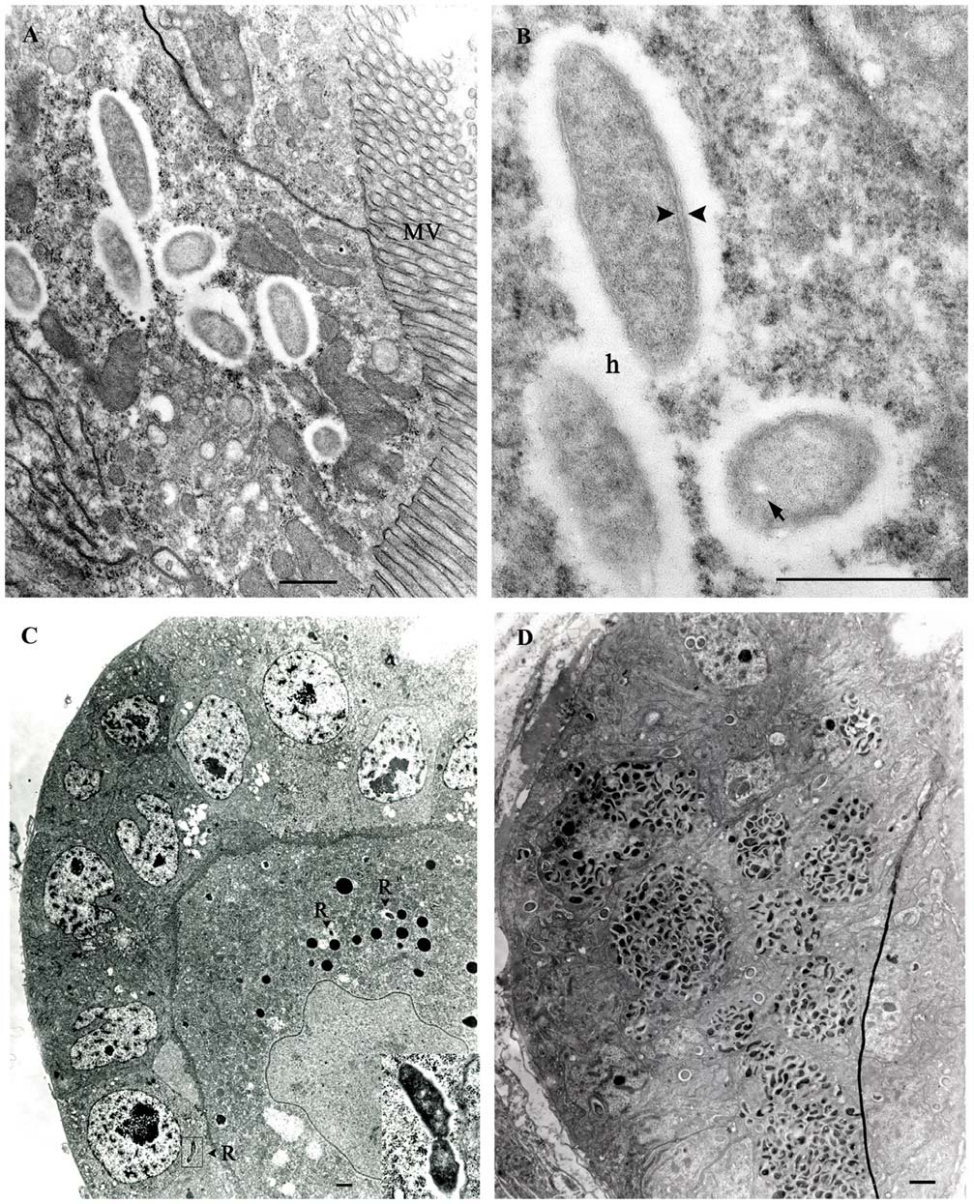
Electron micrographs of *Rickettsia* in booklice. (A) Rickettsiae free in the cytosol of gut epithelial cells. MV represents microvilli. (B) Higher magnification of Fig. 2A shows typical rickettsial ultrastructure: rickettsial cell wall including trilaminar cell wall membrane associated with the external surface microcapsule layer and an internal trilaminar cytoplasmic membrane (arrowheads), surrounded by an outermost “halo” zone (h). Solid arrow indicates a small vacuole inside rickettsial cytoplasm. (C) Rickettsiae (R) in ovary. Inset: higher magnification view of the boxed rickettsiae. (D) Ultrathin section of a mycetome-like, several cells of which contain large vacuoles tightly packed with rickettsiae of irregular shape with dense cytoplasm. Bar = 500 nm.

In order to investigate the presence of *Rickettsia* in booklice associated with the regular maintenance of the LSU colony of cat fleas, formalin-fixed paraffin-embedded booklice sections were stained with polyclonal antibody against *R. felis* followed by a secondary FITC-conjugated antibody. IFA results showed that each booklouse assessed was heavily infected with rickettsiae (Fig. 1A). Numerous rickettsiae were broadly distributed throughout the entire insect body including the head, thorax, and abdomen. Some rickettsiae were dispersed freely in the midgut lumen and body cavity, while others were cell-associated within midgut epithelial cells, fat body cells, and ganglia. Several spherical or elongated mycetomes containing a number of globular mycetocytes densely packed with smaller rickettsiae were observed primarily in the abdominal portions of the booklice (Fig. 1C–F). A similar pattern of mycetomes of booklice in which globular mycetocytes containing compact and slightly smaller rickettsiae was previously described by Perotti *et al.* [6]. The presence of *Rickettsia* in booklice was also examined by TEM. Rickettsiae with typical ultrastructure were identified throughout booklice tissues including gut, ovary, salivary gland, and mycetome (Fig. 2). In the gut, rickettsiae were found freely in the cytosol (Fig. 2A); and, under higher magnification (Fig. 2B), distinctive morphology of *Rickettsia* including a trilaminar cell wall associated with the external surface microcapsular layer and an internal trilaminar cytoplasmic membrane surrounding the cytoplasm (arrowheads) was evident. Adjacent to the microcapsule, rickettsiae appeared to be surrounded by a clear outermost layer termed the “halo” zone (h) [20]. These features are very characteristic for rickettsiae and absent in *Wolbachia*. Some rickettsiae were observed to contain electron-lucent vacuoles inside rickettsial cytoplasm as described in other *R. felis* isolates [21], [22]. Intact and dividing rickettsiae were found in booklice ovaries (Fig. 2C, inset). Interestingly, a number of globular clusters of densely packed rickettsiae was observed (Fig. 2D). This unique formation was similar to a formation of mycetomes detected by IFA (Fig. 1F). However some of these rickettsiae were enclosed in membrane-bound vacuoles and appeared to have irregular morphology with very dense cytoplasm and enlarged periplasmic spaces which are features characteristic of rickettsial degradation in phagolysosomes.

### Isolation and propagation of *Rickettsia* from booklice

**Figure 3:**
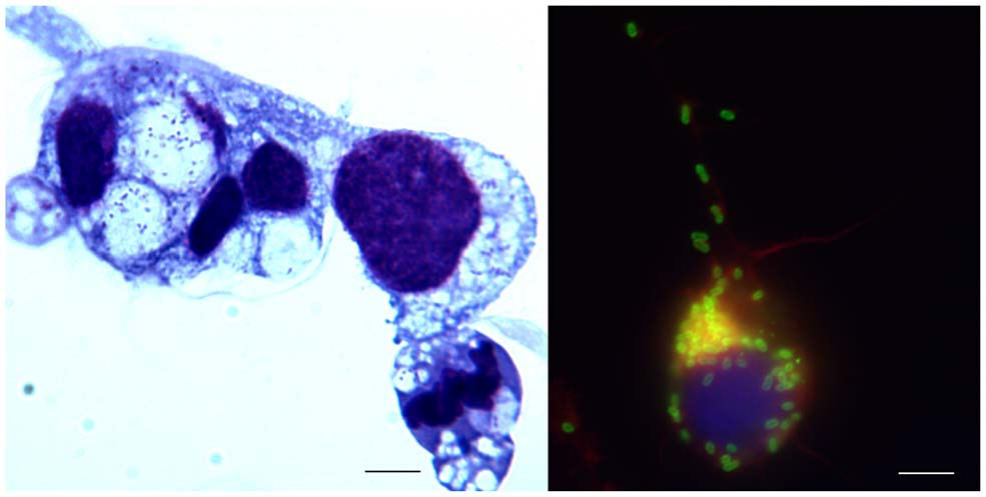
Propagation of *Rickettsia* isolated from booklice in ISE6 cells. (A) ISE6 cells infected with *Rickettsia* isolated from booklice were Cytospin centrifuged and rickettsiae were visualized by Diff-Quik staining. (B) The infected cells on coverslips were fixed and permeabilized prior to stain with mouse polyclonal antibody against *R. felis* and FITC-conjugated goat anti-mouse IgG. Host cell actin and nuclei were stained with with Rhodamine-Phalloidin and DAPI as shown in red and blue, respectively. Bar = 5 µm.

Booklice were sequentially washed and inoculated into flasks of ISE6 cells. Even in the absence of grinding, the process of cleaning and transferring to cell growth medium resulted in maceration of booklice. Of the two pools, the pool that was further ground acquired fungal infection by 14 days post-inoculation (dpi) and was discarded. The second pool of booklice was free of outside contamination and PCR verified the presence of rickettsial DNA in the culture (data not shown). Microscopic detection of rickettsiae was possible at 21 dpi and identified typical-shaped rickettsiae inside host cells with some cells containing large numbers of individual organisms (Fig. 3).

### Microscopic characterization of *L. bostrychophila* rickettsial isolate in ISE6 cells

**Figure 4:**
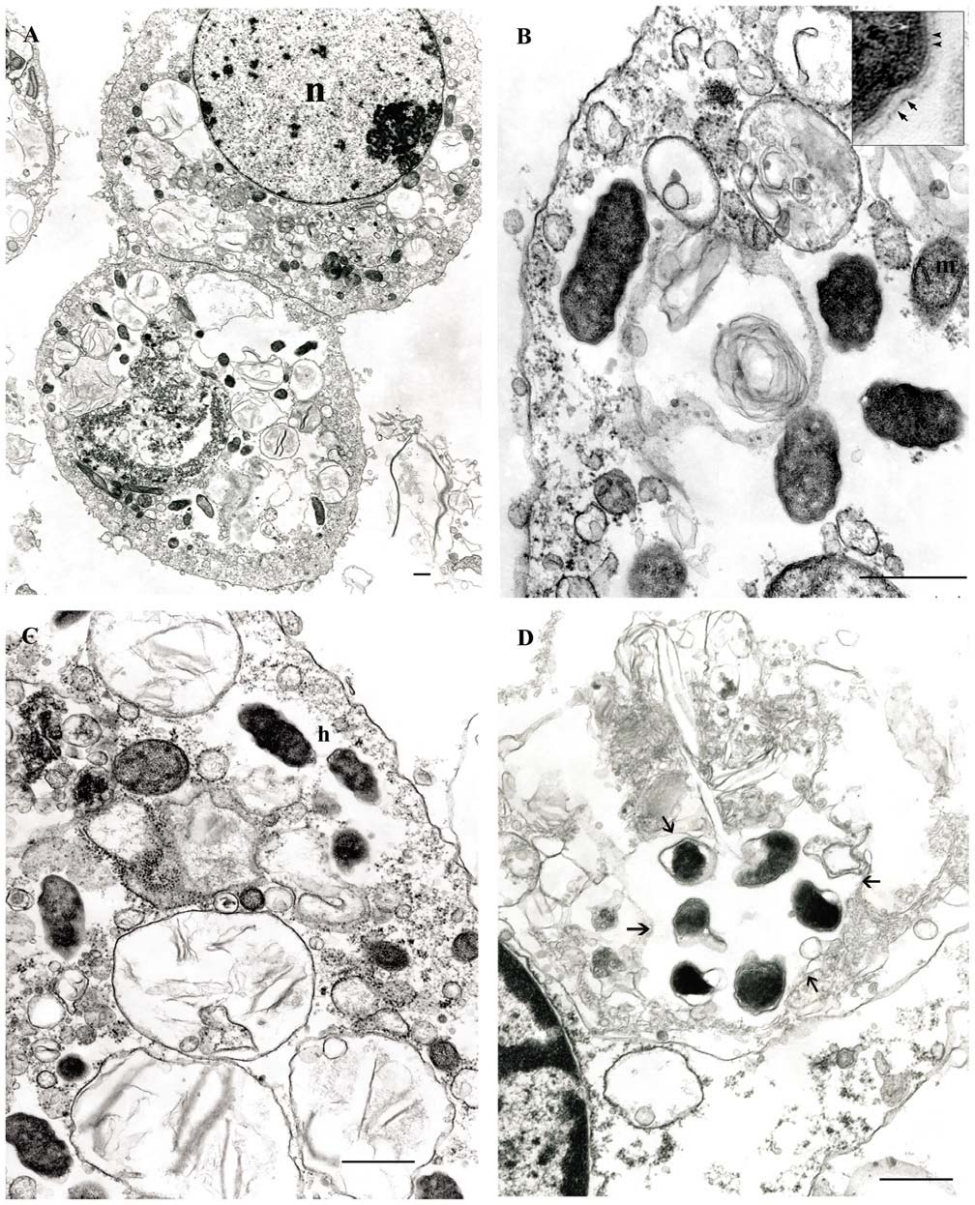
Electron micrographs of *Rickettsia* isolated from booklice in ISE6 cells. (A) Typical infected cells containing rickettsiae in cytosol. (B) Typical ultrastructure of *Rickettsia*. Inset: higher magnification view of rickettsial cell wall including trilaminar cell wall associated with the external surface microcapsule layer (solid arrows) and an internal trilaminar cytoplasmic membrane (arrowheads). m represents mitochondria. (C) Rickettsiae surrounded by a halo zone (h). (D) Rickettsiae being destroyed in a phagolysosome. Long arrows indicate phagolysosomal membrane. Bar = 500 nm.

The ultrastructure of *Rickettsia*-infected (passage 3) ISE6 cells was examined by TEM at 19 dpi. Infected cells were observed containing typical rickettsiae which were found free in cytosol within large spaces devoid of organelles (Fig. 4A). Higher magnification (Fig. 4B) identified typical morphology of *Rickettsia* as described in booklice (Fig. 2A–B) including the external surface microcapsular layer (inset; solid arrows) and an internal cytoplasmic membrane surrounding the cytoplasm (inset; arrowheads) and surrounded by the halo zone (h, Fig. 4C). Similar to the observation in booklice, morphologically distinct rickettsiae with very dense cytoplasm and enlarged periplasmic spaces were occasionally identified in membrane-bound vacuoles, suggesting that they were being destroyed in phagolysosomes (long arrows, Fig. 4D).

### Molecular characterization of *L. bostrychophila* rickettsial isolate

The six DNA sequences from the *L. bostrychophila* rickettsial isolate cultivated in ISE6 cells confirmed *R. felis* identity via the sequencing of a portion of genes from *R. felis* genome including *gltA*, *Rickettsia* specific 17-kDa antigen, and *ompA*; and, the *R. felis* plasmid including a pRF diagnostic region and pRF putative *tldD/pmbA*. These sequences from the new *R. felis* LSU-Lb isolate were compared to those of *R. felis* URRWXcal2 genome or plasmid pRF sequences available on GenBank (accession numbers CP000053 and CP000054, respectively) and their identity was summarized in Table 1. Previous nucleotide sequence analysis for several genes from *R. felis* LSU (flea isolate) [15] were identical to *R. felis* URRWXcal2 and therefore comparison to *R. felis* LSU are the same. Nucleotide sequences for both the *gltA* (341 bp) and *Rickettsia* specific 17-kDa antigen gene (394 bp) were identical to the *R. felis* URRWXcal2 genome sequences. Amplification of the ORF for *ompA* (3816 bp) revealed a sequence 99% identical (a single nucleotide difference) to URRWXcal2 genome sequence and our *R. felis* LSU cat flea isolate *ompA* sequence (GenBank accession number DQ408668). Two plasmids, pRF and pRFδ, were identified in the type strain of *R. felis* (URRWXcal2) [17]. For the current isolate, a 112 bp portion of the *R. felis* plasmid pRF that was identical to *R. felis* URRWXcal2 plasmid was amplified. Similar to the *R. felis* LSU flea isolate, the portion of the smaller plasmid, pRFδ, targeted with primer set pRFa and pRFd did not result in amplification, suggesting the absence of pRFδ. Recent genetic characterization of *R. felis* pRF in *L. bostrychophila* suggested a unique sequence for an *R. felis*-specific putative *tldD/pmbA* (accession number GQ329881) containing a single nucleotide mismatch compared to URRWXcal2 plasmid sequence [13]. In the current study, two different nucleotide sequences of 461 bp were amplified from this portion of the plasmid for *R. felis* LSU-Lb. In addition to the sequence with 100% (461/461) identity to URRWXcal2 plasmid sequence and *R. felis* pRF in booklice [13], a second sequence containing only 90% (414/461) nucleotide identity to the previously published sequences was identified.

**Table 1:** Identity of sequenced *R. felis* LSU-Lb isolate genes compared to *R. felis* URRWXcal2 Accession numbers CP000053 (genome) and CP000054 (plasmid pRF).

Gene sequenced	Matching nucleotides	Identity
Citrate synthase gene (*gltA*)	341/341	100%
17-kDa surface antigen gene	394/394	100%
pRF diagnostic region	112/112	100%
pRF putative *tldD/pmbA*	414/461, 461/461	90% or 100% (Two sequences)
*ompA*	3816/3817	99%

**Figure 5:**
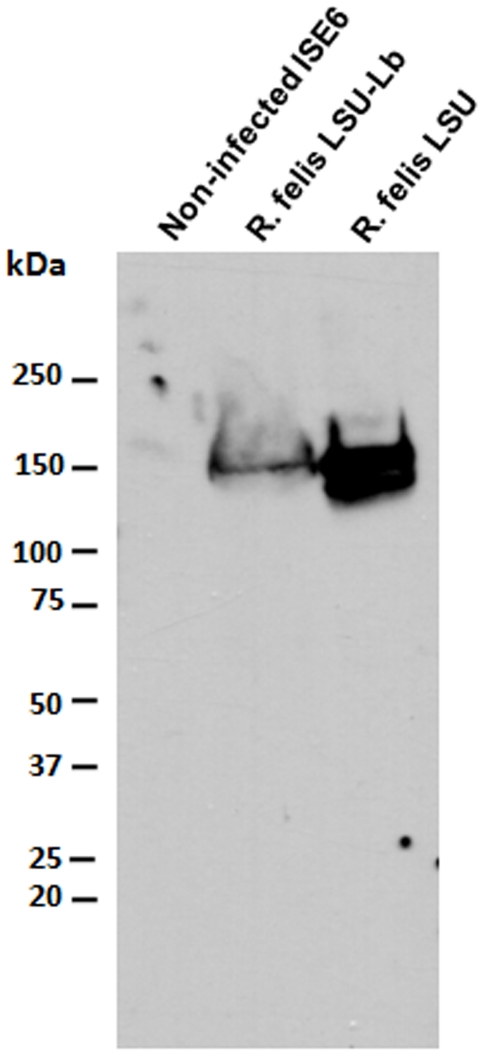
Rickettsial OmpB antigen detected in the *L. bostrychophila* isolate. Rickettsial proteins were extracted from partially purified LSU or LSU-Lb *R. felis* isolates. Rickettsial antigen was detected by Western immunoblotting using an anti-rickettsial OmpB monoclonal antibody. The reactive bands represent the *R. felis* OmpB protein. Protein extracted from non-infected ISE6 cells was served as a negative control.

The isolate was confirmed using Western immunobloting analysis of rickettsial proteins with monoclonal antibodies directed against the common rickettsial outer membrane protein B (OmpB). Comparison of low-passage isolates confirms that *Rickettsia* was present in the culture (Fig. 5). A similar banding pattern, as the one observed for *R. felis* (LSU), was determined to be OmpB as verified by mass spectrometry in a previous study [19].

### Comparative rickettsial loads in insects

**Figure 6:**
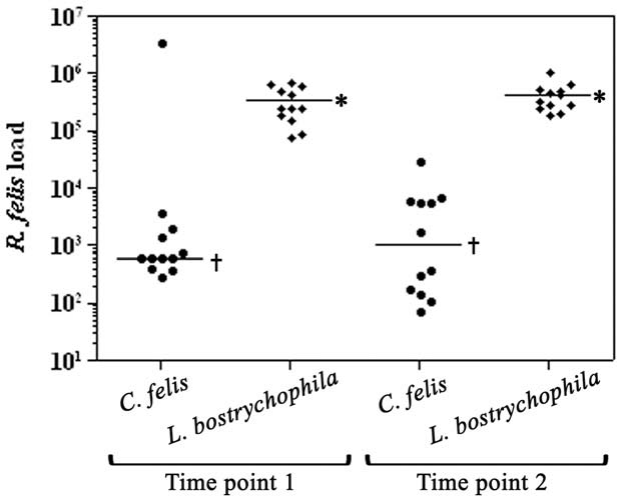
Rickettsial load in individual *L. bostrychophila* and *C. felis* (LSU colony). Twelve individual booklice and cat fleas were collected at two time points (June 2010 and October 2010). Rickettsial loads were determined by qPCR. Scatter plot shows significantly greater rickettsial loads in *L. bostrychophila*, compared to *C. felis*. Bars represent medians of the data set. The medians with the same symbol are not significantly different (*p*≤0.05).

To evaluate the number of rickettsiae in individual booklice compared to cat fleas (LSU), quantities of rickettsiae were determined by qPCR from gDNA extracted from twelve individual booklice or cat fleas. Rickettsial loads were evaluated at two different time points (June 2010 and October 2010) to accurately capture the rickettsial load in multiple generations in each insect. The median rickettsial load of booklice collected in June and October 2010 were 2.4×10^5^ (range: 7.4×10^4^–6.7×10^5^) and 3.8×10^5^ (range: 1.9×10^5^–1.0×10^6^) rickettsiae per booklouse, respectively. Cat fleas contained relatively less rickettsiae with 5.9×10^2^ (range: 2.8×10^2^–3.5×10^3^) and 1.0×10^3^ (range: 7.2×10^1^–6.7×10^3^) rickettsiae per flea from the two collection timepoints (Fig. 6). With the exception of a single cat flea with a heavy infection load (3.4×10^6^ rickettsiae), the median rickettsial loads in booklice were significantly greater compared to the cat flea (*p*<0.05) at both time points assessed.

## Discussion

Currently, there are three flea-derived *R. felis* isolates available for study [14]–[16]. With diverse arthropod hosts, unique culture characteristics and distinct plasmid content [18], [23], [24], *R. felis* is truly a dynamic *Rickettsia*. In the current study, we add to the isolate repertoire by describing a new *R. felis* isolate (strain LSU-Lb) from *L. bostrychophila*, an insect only recently recognized as a host for *R. felis* [13]. Furthermore, detailed with ultrastructural analysis and quantitative biological measurements were provided in order to begin to unravel the ecology of *R. felis*.

Rickettsial endosymbionts were first identified in the common booklouse, *L. bostrychophila*, using PCR to amplify a portion of the 16S rRNA gene, providing indication of rickettsial origin, but not specific designation [11]. Fluorescence *in situ* hybridization (FISH) was employed for a thorough characterization of the distribution of rickettsiae in *L. bostrychophila* identifying widespread infection of the insect with heavy infection in oocytes and in myceteomes [6]. Additionally, mycetomic *Rickettsia* were found to be associated with booklice development, parthenogenesis, and are maintained by transovarial transmission [6]. Using a different approach in the current study, a monospecific polyclonal antibody to *R. felis* (LSU) was used to detect systemic rickettsial infection naturally occurring in *L. bostrychophila*. Similar to FISH analysis, mycetomes varying in number and shape and containing intense signal of rickettsiae in globular mycetocytes were observed. Mycetomic rickettsiae in booklice migrate to multiple sites during the developmental stages of booklice through several tissues *e.g.* fat body, midgut, ovaries, and finally form a pair of mycetomes located between the midgut and ovaries in the adult stage [6]. A mixed life cycle stage population of booklice was collected for use in this study leading to the observed *Rickettsia* throughout the booklice with variation in number and localization of mycetomes as determined by the intense IFA signal. Detection of rickettsiae by TEM in the ovaries, specifically dividing organisms in the oocytes, strongly favors their transovarial transmission. Likewise, the transovarial transmission of rickttsiae has been observed in other populations of *L. bostrychophila* [6] and in colonized *C. felis* [25], [26]. Adding to the known distribution of rickettsiae in insects, the current study provides novel ultrastructural analyses of *R. felis* in the arthropod host.

To further explore the biology of *L. bostrychophila*-associated *Rickettsia*, an isolate of the organisms from whole booklice was made using a well-defined tick cell culture system [27]. Microscopic examination demonstrated typical rickettsiae with morphology consistent with flea-borne *R. felis* isolate [15]. Individual tick cells were observed to become heavily infected and the infection produced a sponge-like appearance in host cells, similar to the cat flea isolate [15]. Also, similar to other *Rickettsia*-tick cell models [27], cells appear to be able to tolerate high numbers of rickettsiae in the absence of cell lysis. Ultrastructural analysis of this newly isolated *R. felis* (LSU-Lb) in ISE6 cells is consistent with previous examination of *R. felis* (LSU) from cat fleas. The isolated *R. felis* (LSU-Lb)-infected cells were found intact containing free rickettsiae in cytosol without a clear appearance of vacuolization. Similar to *R. felis* detected in cat fleas salivary glands [22], irregular-shaped dense rickettsiae were apparently being destroyed in a membrane-bound vacuole, which is a characteristic of phagolysosomal lysis. Ultrastructural observation revealing rickettsiae undergoing degradation in phagolysosomes were previously reported (as putative autophagolysosomes) in other *Rickettsia*-host interactions including *Rickettsia peacockii* in *Dermacentor andersoni* [28] and *Rickettsia conorii* in mouse endothelial cells [29]. The lysis of *R. conorii* in double-membraned vacuoles in mouse endothelium was dependent on nitric oxide synthesis and known as an autophagic pathway which helps regulate the amount of intracellular rickettsiae and facilitates cell survival [29]. This process may help the cell tolerate a moderate amount of rickettsiae and support a persistent infection. Interestingly, in booklice rickettsiae were being degraded in the mycetome-like formation as observed by TEM. The potential interplay is noteworthy as rickettsial endosymbionts are essential for booklice parthenogenesis [6]; and, yet, to some extent they appear to be destroyed by the host. While degeneration of endosymbionts by the insect host to control the symbiotic interaction has been described [30], this interaction requires further examination in the *R. felis*/arthropod host system.

Subsequent to the initial identification of rickettsial endosymbionts in booklice, and phylogenetic placement of *R. felis* with the booklouse rickettsial strain [4], thorough molecular characterization of the *Rickettsia* definitively defined the species [13]. It was demonstrated that the 63 kb plasmid (pRF) described in the type *R. felis* isolate [17] was also present in *L. bostrychophila* and identical by nucleotide assessment. Distinct from the type strain, but similar to the cat flea *R. felis* (LSU) isolate [15], the smaller plasmid, pRFδ, was not detected in *L. bostrychophila*. The *R. felis* (LSU) cat flea isolate has an estimated six copies of pRF per genomic equivalent [17]; however, full plasmid content of the *R. felis* LSU-Lb isolate was not assessed in the current study. Other gene portions assessed are consistent with database sequences for cat flea-derived isolates of *R. felis*. Interestingly, the pRF putative *tldD/pmbA* amplicon from the *R. felis* isolate LSU-Lb produced two gene sequences, one identical and one that was 10% different in nucleotide identity, compared to the cat flea-derived isolate [17] and the recent molecular characterization of the pRF from *L. bostrychophila* [13]. These two genotypes were consistently amplified, suggesting the potential unique gene sequences on different plasmid copies and possibly providing differentiation tools. It is recognized that there are multiple plasmid copies in *R. felis*, with different sequences for some genes such as rickettsial heat shock protein genes [17], and further genetic analysis of this new isolate is needed.

Rickettsial load in booklice quantified by qPCR corresponded to the intense rickettsial infection detected by IFA in the same population of booklice. Previous quantitative analyses of rickettsial load in naturally infected cat fleas demonstrated large variability in numbers of rickettsiae present in individual cat fleas, ranging from 1.3×10^3^ to 1.6×10^7^ [31]. In the current study, a survey of twelve infected cat fleas from unique generations four months apart identified a much lower rickettsial load for most of the samples, consistent with the low-end range of the previous study. Conversely, the mean rickettsial load in an individual booklouse was determined to contain a steady level of *R. felis* significantly greater than what was typically observed in cat fleas. Given the smaller size of booklice, compared to cat fleas, the data suggest that individual booklice carry a greater rickettsial burden compared to its hematophagous counterparts. While limited variability in rickettsial load was observed in cat flea populations during feeding [31], the rickettsial load should be monitored in each lifecycle stage for both hosts to determine if differential growth kinetics are a component of host-specific transmission.

Of particular interest is the transmission potential of *R. felis* between invertebrates and to vertebrate hosts. It has been suggested that the relatedness of transitional group member *R. felis* to *Rickettsia* species in non-hematophagous arthropods limits the potential for transmission of *R. felis* from vertebrate hosts to arthropod vector [4]. In association with the recent molecular characterization of *R. felis* in *L. bostrychophila*, it was noted that the shared microhabitat between fleas and *L. bostrychophila* and the phoretic relationship of *R. felis*-infected *L. bostrychophila* with vertebrate hosts facilitates the horizontal transmission of *R. felis* from fleas to *L. bostrychophila* [13]. Given the ability of *L. bostrychophila* to consume insect eggs [32], this mode of transmission is quite possible. Based on the system utilized for the current study, a different scenario may transpire by which fleas acquire new *R. felis* infection via consumption of *L. bostrychophila*. The LSU colony of cat fleas is maintained on shorthair cats which utilize shredded corn cob shavings in the litter box; the same shavings are the origin of the *L. bostrychophila* used for isolation of *R. felis* in the current study. On regular intervals, corn cob shavings and potentially booklice are dislodged from the litter box and are collected with the cat flea eggs. While the large particles are sifted out via a 40µm filter, cat flea eggs and *L. bostrychophila* or *L. bostrychophila* eggs are collected and maintained together with cat flea eggs for colony maintenance. The cannibalistic nature of cat flea larvae from the LSU colony has been previously examined [33]. Larval cat fleas require nutrients including adult flea feces and flea eggs as a component of successful development to the adult stage [33], and would consume *L. bostrychophila*. This alternative hypothesis is also supported by the well-documented variability, ranging from 35 to 100%, in *R. felis* incidence in the LSU cat flea colony [1]. While there is known vertical transmission in both non-animal host colonized cat fleas [26] and in the LSU colony of cat fleas [25], the efficiency of vertical transmission is not 100%. Regular feeding of LSU colonized fleas on non-vertebrate hosts (and *L. bostrychophila*-free rearing conditions) results in decreased vertical transmission of *R. felis* infection over twelve subsequent generations [25]. In contrast to the symbiotic relationship and stable vertical transmission associated with *Rickettsia* infection of *L. bostrychophila* [6], a more transient relationship between cat fleas and *R. felis* requires steady introduction of *R. felis* into flea populations. Larval cat flea consumption of *L. bostrychophila* eggs or immature insects could serve as this steady source of infection. Once inside a host, such as a cat flea, *R. felis* infection is transient and limited vertical transmission efficiency requires, to some extent, a horizontal transmission event.

The complexity of *R. felis* relating to a host both intrigues and provides several research opportunities. For example, the concurrence of these two populations and the incidence of *R. felis* in cat fleas should be further explored. Similar to the flea-borne infection model for *Rickettsia typhi* [34], infection of adult cat fleas with *R. felis* via an infectious bloodmeal is possible; however, vertical transmission of the newly acquired *R. felis* infection is not observed [35]. Additionally, the presence of other endosymbionts (*e.g. Wolbachia* spp.) in booklice has not been clearly established [13]. Using traditional PCR and the wsp-81F [36] and wsp-691R [37] primers we were unable to detect *Wolbachia* DNA in gDNA preparations of either the *L. bostrychophila* or the *L. bostrychophila*-derived isolate of *R. felis* (data not shown); in contrast to observed *Wolbachia* in cat fleas and the flea-derived isolate of *R. felis* [19], [38]. The influence of *Wolbachia* on the incidence of *R. felis* in cat fleas has been surveyed [38], but the direct interaction between the two bacteria in the arthropod host remains to be examined thoroughly. The maintenance of a *L. bostrychophila* colony and a *C. felis* colony and both respective *R. felis* isolates will facilitate development of bioassays to test hypotheses regarding *R. felis* pathogenicity and transmission. In addition to deciphering the mechanisms of transmission between arthropod and vertebrate hosts, the differential expression of rickettsial factors between non-hematophagous and hematophagous arthropod hosts will enable a better understanding of the molecular determinants of rickettsial pathogenicity.

## Materials and Methods

### Source of booklice and fleas

*L. bostrychophila* were initially collected from cat flea rearing containers and identified at the Louisiana State Arthropod Museum. The source of booklice was determined to be shredded corn cobs (Harlan Laboratories) used in litter trays for the cats used in cat flea maintenance. Booklice were recovered regularly from shredded corn cobs held in containers with fine Caribbean sand and flea larval diet, in the absence of fleas. Maintenance of *C. felis* at the Louisiana State University School of Veterinary Medicine (LSU-SVM) on domestic shorthair cats was as previously described [39].

### Cell culture and isolation of *Rickettsia*

*Ixodes scapularis*-derived ISE6 cells, provided by T. Kurtti (University of Minnesota), were maintained in antibiotic-free L15B growth medium supplemented with 10% heat-inactivated fetal bovine serum (HyClone), 10% tryptose phosphate broth (Becton, Dickinson and Company) [40], at pH 6.8–7.0 in a humidified 5% CO_2_ incubator at 32°C. For rickettsial isolation, two pools of 25 *L. bostrychophila* were sequentially washed over filter paper as follows: three times in 70% ethanol, one time in 10% sodium hypochlorite, and rinsed in sterile distilled water. Booklice pools were either transferred by pipette tip to a sterile glass pestle tissue grinder and ground in 25 µl of L15B tick cell culture media or directly transferred to a 25-cm^2^ tissue culture flask containing ISE6 cells (passage 134). ISE6 cells with booklice preparations were immediately placed at 32°C. For regular maintenance of *Rickettsia*-infected cells, they were passed at a ratio of 1∶5 every 21 to 28 days. Upon each passage of ISE6 cells exposed to booklice homogenates, a portion of the cells were prepared for staining using a Cytospin centrifuge (Wescor) and rickettsial infection was assessed by traditional PCR [15] and/or Diff-Quik (Dade Behring) staining, according to the manufacturer's protocol.

### Microscopy

For transmission electron microscopy (TEM), a portion of a 25-cm^2^ flask with 90% of the ISE6 cells infected with third passage rickettsiae (assessed by Cytospin) was dislodged and collected by centrifugation in a 1.5 ml microcentrifuge tube. After removing growth medium and covering the cell pellet with phosphate buffered saline (PBS), the cell pellet was transported to University of Texas Medical Branch (UTMB) on ice and resuspended in fixative containing 2.5% formaldehyde, 0.1% glutaraldehyde, 0.03% CaCl_2_, and 0.03% trinitrophenol in 0.05 M cacodylate buffer, pH 7.4 [41]. The sample was post-fixed in 1% osmium tetroxide in 0.05 M cacodylate buffer (pH 7.2), stained en bloc for 20 min with 2% aqueous uranyl acetate at 60°C, dehydrated in a graded series of ethanol, and embedded in Poly/Bed 812 (Polysciences). Whole booklice with the heads removed were immersed in the same primary fixative for 24 h, transported to UTMB in PBS on ice, then post-fixed and processed in the same way as the cell cultures except, in addition to Poly/Bed embedding, several booklice were also embedded in a low viscosity Spurr resin. Semi-thin and ultrathin sections were cut on a Leica UC7 ultramicrotome. Semi-thin sections were stained with toluidine blue and ultrathin sections stained with lead citrate and examined in a Philips 201 transmission electron microscope at 60kV as previously described [21].

For immunofluorescence assay (IFA), slides containing formalin-fixed paraffin-embedded booklice sections were heated at 65°C for 15 min and deparaffinized by several immersions in Hemo-De, a Xylene substitute (Scientific Safety Solvents). Slides were rinsed with PBS prior to antigen retrieval treatment with 10 µg/ml proteinase K (Roche) in pre-warmed buffer containing 1 M Tris and 0.5 M EDTA (pH 8) at 37°C for 15 min. The treated slides were extensively washed with PBS prior to immunofluorescence staining. For *R. felis* detection, a polyclonal antibody against *R. felis* was generated in mice as described by Sunyakumthorn [19]. The pretreated slides were blocked with 3% bovine serum albumin (BSA) in PBS for 1 h prior to incubation with mouse polyclonal anti-*R. felis* at a dilution of 1∶100 in 1% BSA in PBS for 2 h. After incubation, slides were washed three times with PBS containing 0.01% Triton X-100 and incubated with FITC-conjugated goat anti-mouse IgG (KPL) at a dilution of 1∶200 for 1 h. After being washed as above, booklice tissues were counterstained with 0.1% Evan's blue in PBS at 37°C for 30 min. All incubation steps were completed in a humidified chamber. Cover slips were mounted with VECTASHIELD ® Hard Set™ (Vector Laboratories Inc.) in the presence of DAPI (4′, 6-diamidino-2-phenylindole) for nuclear counterstaining. Then slides were viewed under a fluorescence microscope. Booklice sections stained with non-infected mouse serum followed by secondary FITC-conjugated goat anti-mouse IgG served as a negative control.

### DNA isolation and PCR amplification

Genomic DNA (gDNA) of *R. felis* LSU-Lb isolate cultivated in ISE6 cells was extracted using DNeasy blood and tissue kit (QIAGEN) according to the manufacturer's protocol for gram-negative bacteria. PCR reactions utilized gene-specific primers for the *gltA*, *ompA*, *Rickettsia* genus-specific 17-kDa antigen gene, the diagnostic region of the *R. felis* plasmids, pRF and pRFδ, and putative plasmid gene *tldD/pmbA* (Table 2). Reactions were performed using PCR master mix (Promega) or FastStart Master (Roche) and thermocycler conditions previously described [15]. For each set of reactions, an environmental negative control for gDNA extraction (1 µl) and negative control for PCR reaction (1 µl of water) were included. Amplified products were visualized on agarose gels stained with SYBR Safe (Invitrogen).

**Table 2:** Primers used for PCR amplification and sequencing of *R. felis* (LSU-Lb) and primer/probe combination used for quantitative real-time PCR for *Rickettsia*.

Target/Primer name	Nucleotide sequence (5′-3′)	References
**Citrate synthase (** ***gltA*** **)**		
*Rp*CS.877p	GGGGGCCTGCTCACGGCGG	[43]
*Rp*CS.1258n	ATTGCAAAAAGTACAGTGAACA	
**17-kDa genus-specific**		
*Rr*17.61p	GCTCTTGCAACTTCTATGTT	[44]
*Rr*17.492n	CATTGTTCGTCAGGTTGGCG	
**190-kDa antigen (** ***ompA*** **)**		
*Rf190.1fw*	ATGGCGAATATTTCTCTAAAATTA	[45]
*Rf190.1800rev*	TTAACTCACCACCACCGTTAGCAAGACCG	
*Rf. ompA-862fw*	CAGCAATAAGTGTAGGAGCA	This Study
*Rf. ompA-1337fw*	CGGAGTAGTAAAAGCGAATG	
*Rf. ompA-1836fw*	TAACGGTGGTGGTGAGTTA	
*Rf. ompA-2334fw*	CAAGTGTCGGAAATGCTACT	
*Rf. ompA-2862fw*	GGTAGTGTTAGCGGTGTTGT	
*Rf. ompA-3792rev*	GCCGGGCTGTATTGCAT	
*Rf. ompA-3348fw*	ATAAGCCCGGATACAAAATA	[15]
*Rf190.553fw*	CTTGCAGGAAATATAGATGGAG	
***R. felis*** ** plasmid(s)**		
pRFa	CAAGCTTTTGTACTGCCTCTAT	[17]
pRFb	AGTGCATATAGCTACCACACTATCT	
pRFd	GCTTATGTTCGCCTTTAGTATTTA	
***tldD/pmbA*** ** plasmid gene**		
pRF *tldD/pmbA*-1fw	TTAAAATCTCATCGTCATTGT	This Study
pRF *tldD/pmbA*-501rev	ATGTTAACTGCAATTCGCC	
**qPCR**		
*Rickettsia* forward	ATGAATAAACAAGGKACNGGHACAC	[42]
*Rickettsia* reverse	AAGTAATGCRCCTACACCTACTC	
probe (FAM-BHQ)	CGCGACCCGAATTGAGAACCAAGTAATGCGTCGCG	

*IUB codes for mixed bases at a given position: N = A+T+C+G; H = A+T+C; Y = C+T; K = G+T; R = A+G.

### DNA sequencing and analysis

PCR amplicons were purified using Wizard Gel and PCR cleanup system (Promega) according to the manufacturer's protocol. Then, the purified PCR products were cloned into pCR4-TOPO vector (Invitrogen) according to the manufacturer's protocol. At least three clones of each PCR amplicon were sequenced by the dye terminator method on a 3130 genetic analyzer (Applied Biosystems). Sequencing results were analyzed using the BioEdit sequence alignment editor (Ibis Biosciences); primer sequences were removed and nucleotide similarity comparisons were made using the GenBank database. Distinct nucleotide sequences for *ompA* and putative *tldD/pmbA* were submitted to GenBank under accession numbers HM636635 and HQ003933, respectively.

### Gel electrophoresis and Western immunoblotting

Passage four of *R. felis* (LSU) or *R. felis* (LSU-Lb) were partially purified from infected ISE6 cells using 27-gauge needle lysis followed by high speed centrifugation at 13,400×*g* for 10 min at 4°C. Rickettsial pellets and a cell pellet from uninfected ISE6 cells were resuspended and incubated in urea/thiourea lysis buffer [8 M urea, 2 M thiourea, 50 mM dithiothreitol, and 0.4% triton X-100] for 15 min at room temperature. Then, cells were disrupted by sonication for three 5 min intervals. Isolated proteins in the supernatant fraction were recovered by centrifugation at 13,400×*g* for 10 min at 4°C and protein concentrations were determined using a Bradford protein assay (Bio-Rad). Protein extracts (20 to 60 µg of each) were subjected to sodium dodecyl sulfate-polyacrylamide gel electrophoresis (SDS-PAGE) using a 4–15% polyacrylamide precast gel (Bio-Rad). Separated proteins were transferred to a polyvinylidene fluoride membrane (Invitrogen) and blocked with 3% BSA in Tris-buffered saline with 0.1% Tween 20 (TBST) for 1 h at room temperature. The rickettsial protein bands were labeled by incubating membranes with monoclonal antibody against rickettsial OmpB (RC-9C2; Fuller Laboratories) at a dilution of 1∶1,000 in 1% BSA/TBST buffer. After incubation for 2 h, membranes were incubated with horseradish peroxidase-conjugated goat anti-mouse IgG (Sigma) at a dilution of 1∶10,000 in 1% BSA/TBST buffer for 1 h at room temperature. After washing with TBST, the signal was detected using the SuperSignal West Pico Chemiluminescent Substrate Kit (Pierce).

### Quantitative real-time PCR (qPCR)

For individual *C. felis* and *L. bostrychophila*, gDNA was isolated (QIAGEN) and rickettsial load in individual insects was quantitated by qPCR using *Rickettsia*-specific probe and primers selected from a 17-kDa antigen gene consensus sequence specific to *Rickettsia* species [42]. Insects were selected from stock colonies in June and October 2010, representing distinct generations for the populations sampled. Sequences of the degenerate primers (Integrated DNA Technologies) and the probe (Molecular Beacon) are provided in Table 2. The probe was dual-labeled with the reporter FAM and Black Hole Quencher (BHQ). Each 35 µL qPCR reaction was composed of 5 µL of gDNA template, 17.5 µL of 2× LightCycler® 480 Probes Master (Roche), 1 µL of each 10 µM primer, 0.4 µM of probe, and DNase/RNase-free, distilled water. For each reaction, 10 µL were assessed in triplicate on a 384-well plate and amplified using a LightCycler® 480 Real-Time PCR system (Roche). The reactions were pre-incubated at 95°C for 10 min prior to 45 cycles of three step amplification at 95°C for 10 sec, 60°C for 30 sec, and 72°C for 1 sec, followed by cooling down to 40°C for 30 sec. Serial dilutions of the plasmid containing a portion of rickettsial 17-kDa antigen gene were assessed in parallel, serving as a standard for concentration analysis. Quantity of rickettsiae was calculated as copy number of 17-kDa antigen gene per individual insect. Data was statistically analyzed with one-way analysis of variance (Dunn's multiple comparison post test following the Kruskal-Wallis test) using GraphPad Prism version 5.03 (GraphPad Software).
